# Cardiac Computed Tomography-Derived Left Atrial Strain and Volume in Pediatric Patients With Congenital Heart Disease: A Comparative Analysis With Transthoracic Echocardiography

**DOI:** 10.3389/fcvm.2022.870014

**Published:** 2022-06-20

**Authors:** Wei-Hui Xie, Li-Jun Chen, Li-Wei Hu, Rong-Zhen Ouyang, Chen Guo, Ai-Min Sun, Qian Wang, Hai-Sheng Qiu, Yu-Qi Zhang, Hao Zhang, Qi-Hua Fu, Yu-Min Zhong

**Affiliations:** ^1^Department of Radiology, Shanghai Children’s Medical Center, School of Medicine, Shanghai Jiao Tong University, Shanghai, China; ^2^Department of Pediatric Cardiology, Shanghai Children’s Medical Center, School of Medicine, Shanghai Jiao Tong University, Shanghai, China; ^3^Department of Cardiovascular Thoracic Surgery, Shanghai Children’s Medical Center, School of Medicine, Shanghai Jiao Tong University, Shanghai, China; ^4^Pediatric Translational Medicine Institute, Shanghai Children’s Medical Center, School of Medicine, Shanghai Jiao Tong University, Shanghai, China

**Keywords:** cardiac computed tomography, transthoracic echocardiography, LA strain, LA volume, congenital heart disease

## Abstract

**Purpose:**

This study aimed at exploring the feasibility and reproducibility of CCT for the measurement of Left Atrial (LA) strain and volume compared with transthoracic echocardiography (TTE) in pediatric patients with congenital heart disease (CHD).

**Materials and Methods:**

The present study included 43 postoperative patients with CHD (7.39 ± 3.64 years, 56% male) who underwent clinically indicated CCT, and all patients underwent additional TTE on the same day. LA strain and volume parameters were measured by dedicated software. The correlation and agreement of LA strain and volume parameters were assessed using Pearson’s correlation coefficient and Bland-Altman analysis. Intra-class correlation coefficients (ICC) were used to assess CCT intra-observer and inter-observer reproducibility.

**Results:**

All strain parameters of CCT were lower compared to TTE (reservoir strain: 28.37 ± 6.92 *vs.* 32.15 ± 8.15, respectively; conduit strain: 21.33 ± 6.46 *vs.* 24.23 ± 7.75, respectively; booster strain: 7.04 ± 2.74 *vs.* 7.92 ± 3.56). While the volume parameters of CCT were higher compared to TTE (LAV: 29.60 ± 19.01 *vs.* 25.66 ± 17.60, respectively; LAVi: 30.36 ± 22.31 *vs.* 28.63 ± 19.25, respectively). Both LA strain and volume measurements showed good correlation and agreement between the two modalities (*r* = 0.63–0.87, *p* < 0.001). CT-derived LA strain and volume measurements showed good intra- and inter-observer reproducibility using prototype software (*ICC* = 0.78–0.96).

**Conclusions:**

CCT was feasible for measuring LA strain and volume with good correlation and high reproducibility as compared with TTE. As a complementary modality, CCT can regard as an accepted method in the evaluation of LA function in pediatric patients with CHD

## Introduction

The left atrial (LA) function has recently emerged as a powerful parameter (1). The quantification of LA structure and function can identify the presence of subclinical atrial disease and predicts incident heart failure events among asymptomatic individuals and in the general population (2). Some studies have shown that LA size and function were related to left ventricular (LV) diastolic dysfunction, atrial tachyarrhythmias, and cardiovascular risk burden in pediatric patients with congenital heart disease (CHD) (3–5). Thus, the early detection is important to prevent the development of heart failure symptoms.

The LA function can be separated into three phases: reservoir function (serving as a reservoir to collect pulmonary venous blood in LV systole), conduit function (serving as a conduit for the LA empties passively during early LV diastole), and booster function (serving as a booster pump to augment LV filling during late LV diastole) (6, 7). Dysfunction of these normal LA mechanics will reduce overall cardiovascular function and has been implicated in the development of heart failure with preserved ejection fraction (8). LA volume (LAV) has been shown to provide an index of cardiovascular risk and has improved prognostic and diagnostic information in the assessment of LV diastolic function (9, 10). However, recent studies have found that, as a new LA functional parameter, LA strain measurements may be more sensitive as an early marker of diastolic dysfunction and these measurements have been shown to aid in the diagnosis of heart failure and be accurate predictors of cardiac pressure, exercise performance, and clinical outcomes, such as cardiac hospitalizations and mortality (11–15). Based on three phases of LA function, the LA strain measurements include reservoir strain (reflective of LA reservoir function), conduit strain (reflective of LA conduit function), and booster strain (reflective of LA booster function).

At present, the first-line and most commonly used modality to assess LA structure and function is transthoracic echocardiography (TTE). Speckle tracking echocardiography (STE) is the most widely used method for strain assessment, which calculates strain by tracking tissue deformation *via* characteristic myocardial speckles frame-by-frame (16). However, TTE has inherent technical limitations in some patients with poor echogenic windows and the geometric assumptions of atrial shape depending on the observer’s level of experience (17, 18). Cardiac magnetic resonance (CMR) has shown to be a reliable method for the evaluation of the atrial volume and function but is limited in some patients with implanted cardiac devices, and cannot cooperate because of the long acquisition time (19, 20). Cardiac computed tomography (CCT) is an emerging non-invasive imaging technique that is increasingly used for the evaluation of cardiac function. Due to the advance in algorithms and post-processing technology, feature tracking (FT) has been allowed for strain assessment using full-beat CCT data. Similar to tracking “speckles” with STE, CCT-FT tracking of points or “features” across multiple images based on the pattern-matching techniques. A point is tracked by defining a small patch around the pixel in one frame and finding the most similar patch of pixels in the following image frame allowing motion tracking through successive frames (16). In recent years, there are a growing number of studies on CCT-FT for LA function assessment in adult acquired heart disease and the importance of atrial function is becoming clearer in adults, while the study of CCT-FT in the pediatric population is limited, particularly in postoperative children with CHD (21, 22). Therefore, the purpose of the present study was to analyze the feasibility of CCT to evaluate LA strains and volume in pediatric patients with CHD, assess its reproducibility, and compare it with TTE.

## Materials and Methods

### Study Design and Patient Population

**FIGURE 1:**
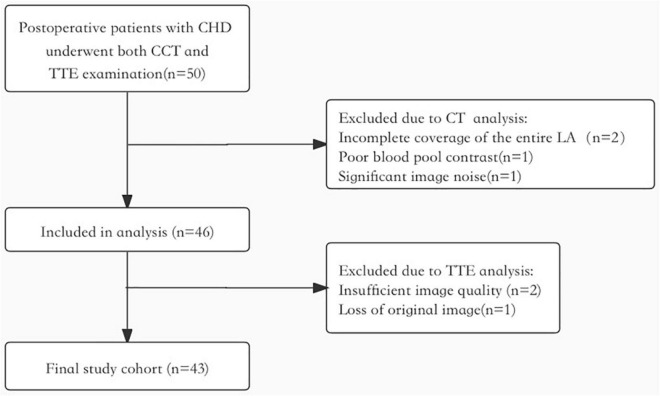
The flowchart of study population.

This was a prospective cross-sectional and single-center study, which consisted of 50 consecutive postoperative patients with CHD with sinus rhythm. For inclusion, patients who underwent clinically indicated follow-up CCT examination from February 2021 to June 2021 were included and transthoracic Doppler echocardiography was performed on the same day. The exclusion criteria were (1) CCT image quality was inadequate for analysis: incomplete coverage of the entire LA (*n* = 2), poor blood pool contrast (*n* = 1), and significant image noise (*n* = 1). (2) Insufficient image quality (*n* = 2) and loss of raw data (*n* = 1). At last, the final study cohort consisted of 43 patients (Figure 1 shows the flowchart of the study population). For this cohort, all children were kept at rest and quiet during the CCT and TTE examinations. Young children < 6 years of age and children who did not cooperate during the examination were sedated with oral chloral hydrate (50 mg/kg) and intravenous pentobarbital (3–5 mg/kg) if necessary. The study was implemented according to the standards of the Declaration of Helsinki and the study was approved by the Shanghai Children’s Medical Center. The patients or patients’ guardians provided written informed consent to participate in this study.

### Cardiac Computed Tomography

#### Cardiac Computed Tomography Acquisition

Cardiac computed tomography was performed using a 16-cm area detector 320-row CT scanner (Aquilion PURE ViSION, Canon Medical, Nasushiobara-shi, Japan) during free-breathing, and each patient underwent a retrospective electrocardiography (ECG)-triggering scan mode. Scanning parameters were as follows: scanning and collimation, 320 mm × 0.5 mm; scan field of view, 25 cm; gantry rotation time, 0.275 s; tube voltage, 80–100 kV; tube current, 29–33 mAs; slice interval, 0.5 mm. The scanning direction was craniocaudal and extended from the level of the thoracic inlet to the diaphragm. A dual-head power injector was used, and non-ionic contrast agent (1.0–1.5 ml/kg; iopamidol, 370 mg/ml, Bracco, Italy) or low-concentration contrast agent (Visipaque, 270 mg/ml, GE Healthcare) was injected through a peripheral vein. The injection rate of contrast medium was 0.8–2.0 ml/s. The bolus-tracking method was used to determine the scan delay, with the region of interest in the descending aorta or left atrium at the level of the carina (the threshold for bolus tracking was 120 HU). The 5–10-ml saline flush technique was applied for all injections to reduce artifacts caused by the undiluted intravascular contrast agents. All scans were reconstructed using a strong adaptive iterative dose reduction algorithm. The CCT dataset was reconstructed in each 5% increment of the R–R interval from early systole (10% of the R–R interval) to late diastole (90% of the R–R interval) and subsequently transferred to dedicated remote workstations enabling further offline analysis.

After cardiac CT scanning, volumetric CT dose index (CTDIvol) and dose length product (DLP) were automatically recorded and stored in our picture archiving and communication system. The effective dose(ED) values of cardiac CT were calculated by the formula: ED = k × DLP, k are the age, sex, and the tube-voltage-specific conversion factors for chest CT according to the International Commission on Radiological Protection (ICRP) publication (23).

#### Image Processing and Data Analysis

**FIGURE 2:**
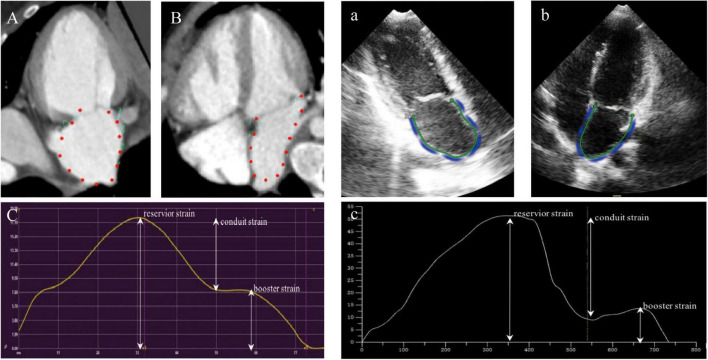
Example for the assessment of LA strain using cardiac computed tomography (CCT) and TTE in two-chamber view **(A,a)**, three-chamber view **(B,b)**, and the strain curve of the result **(C,c)**.

Cardiac computed tomography images were analyzed with the commercial cardiovascular post processing software (QStrain, Medis Suite 3.1, Leiden, Netherlands). The LV parameters were obtained as previously described (24). Two-chamber and four-chamber view focused on LA was rendered. The LA endocardial border was manually traced at the phase of end-diastole and end-systole excluding the LA appendage and the pulmonary veins. The remaining cardiac phases were automatically interpolated. Subsequently, the LA parameters were calculated and got a strain curve of the result (Figure 2). There were two peaks in the strain curve. The first peak corresponded to reservoir strain and the second peak to booster strain, the difference between the two peaks reflected the conduit strain.

### Transthoracic Echocardiography

#### Image Acquisition

Transthoracic echocardiography examination was performed using the Philips iE33 ultrasound machine (Philips, Andover, MA, United States) with a matrix-array transducer (X5-1, X7-2). Children were usually scanned in the left lateral decubitus position. Before the acquisition, echocardiographic images were optimized for the endocardial border visualization. TTE image acquisition was performed in the apical four-chamber view. To encompass the complete LA into the three-dimensional dataset, a full-volume scan (93° × 84°) was acquired in harmonic mode from four R-wave-triggered sub-volumes (93° × 21°). Four cardiac cycles per capture were stitched together. Older children were required to perform breath-holding to avoid respiratory artifacts. After image quality was assured, the dataset was stored on a CD-ROM and transferred to TTE workstations for further analysis.

#### Image Processing and Data Analysis

Transthoracic echocardiography images were analyzed offline using commercially available software (TomTec 4D LV analysis 3.1; TomTec Imaging Systems GMBH, Unterschleissheim, Germany). The LV also was obtained as previously described (24). The LA endocardial contours were marked in the two-chamber and four-chamber long-axis views with the exclusion of the LA appendage and the pulmonary veins and tracked automatically frame by frame. Adjustment of the contours was performed manually if not satisfactory. Finally, the data of LA strain and volume were automatically obtained by software (Figure 2).

### Intra-Observer and Inter-Observer Reproducibility

To assess the intra- and inter-observer reproducibility of LA measurements by CCT, we chosen 15 patients randomly from the study cohort. For intra-observer reproducibility, a radiologist measured LA strain and volume twice with an interval of 1 week. Two observers were blinded to each other measurements and independently assessed LA strain and volume using the same dataset to determine the inter-observer reproducibility.

### Statistical Analysis

The statistical analyses were performed using SPSS 26.0 (IBM Corp, Armonk, NY, United States) and GraphPad Prism 9 (GraphPad, San Diego, CA, United States). All data are presented as mean ± standard deviation or number (percentage) and range. The Shapiro–Wilk test was used to assess the normality of the distribution of continuous variables. Pearson’s correlation coefficient and the Bland–Altman analysis were used to assess correlation, bias, and 95% limits of agreement for LA measurements between CCT and TTE. The relationship between LA strain parameters and LAV and LVM with CCT and TTE was examined using Pearson’s correlation coefficient and linear regression analysis. The intra- and inter-observer reproducibility of CCT-derived LA strain measurements were assessed using the Bland–Altman analysis and intra-class correlation coefficient (*ICC*). A *p*-value of < 0.05 was considered statistically significant.

## Results

### Patients’ Information

In total, 43 postoperative patients with CHD were included in our study. The demographic and clinical characteristics of the study patients were shown in Table 1. The mean age of the study population was 7.39 ± 3.64 years. A total of 56% of patients were male. The volume CT dose index and dose-length product values of the CCT examinations were 7.32 ± 3.73 and 124.81 ± 66.56 mGy⋅cm, respectively. The effective dose of CCT was 2.23 ± 0.66 mSv. The main types of CHD for postoperative examination were summarized in Table 2; the most common types of CHD were pulmonary atresia with Ventricular Septal Defect (19%) and Tetralogy of Fallot (19%).

**TABLE 1:** Demographics of the patient population.

Patient demographics	*N* = 43
Gender	
Male	24(56)
Female	19 (44)
Age (years)	7.39 ± 3.64
Height (cm)	121.43 ± 21.28
Weight (kg)	26.83 ± 14.17
BSA	0.93 ± 0.14
CTDIvol (mGy)	7.32 ± 3.73
DLP (mGy × cm)	124.81 ± 66.56
ED (mSv)	2.23 ± 0.66

*Data are expressed as mean ± standard deviation or frequency (percentage). BSA, body surface area; CTDIvol, volumetric CT dose index; DLP, dose length product; ED, effective dose.*

**TABLE 2:** Main type of CHD for performance of cardiac computed tomography (CCT).

Cardiac defects	Number of cases (%)
Atrial septal defect	4 (9)
Coarctation of the aorta	4 (9)
Mitral stenosis	1 (2)
Patent truncus arteriosus	1 (2)
Patent ductus arteriousus	1 (2)
Pulmonary atresia with intact ventricular septum	4 (9)
Pulmonary atresia with ventricular septal defect	8 (19)
Pulmonary stenosis	3 (7)
Supravalvular aortic stenosis	1 (2)
Tetralogy of Fallot	8 (19)
Transposition of great arteries	2 (5)
Tricuspid atresia	1 (2)
Ventricular septal defect	5 (13)
Total	43

*Data are expressed as frequency (percentage).*

### Left Ventricular Volumes, Mass and Function

Left ventricular volumes, mass, and function measurements were listed in Table 3. The results of measurement of CCT and TTE were compared to each other. The results of left ventricular end-diastolic volume index (EDVi), end-systolic volume index (ESVi), stroke volume index (SVi), and cardiac output index measured by CCT were significantly higher compared with TTE. However, both LVMASS and LVMASSi in CCT were lower compared with TTE (LVMASS: 34.88 ± 16.43 *vs.* 49.59 ± 17.45, respectively, *p* < 0.001; LVMASSi: 39.06 ± 13.89 *vs.* 58.18 ± 20.66, respectively, *p* < 0.001).

**TABLE 3:** LV volume and mass and function measurements of CCT and TTE.

Measurement	CCT	TTE	*P*-value
LVEF (%)	59.10 ± 6.28	61.85 ± 4.87	0.188
LVEDVi (ml/m^2^)	87.16 ± 33.03	68.02 ± 34.64	< 0.001*
LVESVi (ml/m^2^)	36.38 ± 15.02	23.18 ± 10.22	0.008*
LVSVi (ml/m^2^)	50.56 ± 22.23	44.84 ± 27.13	< 0.001*
LVCOi(l/min/m^2^)	4.21 ± 2.53	3.89 ± 2.97	0.134
LVMASS(g)	34.88 ± 16.43	49.59 ± 17.45	< 0.001*
LVMASSi(g/m^2^)	39.06 ± 13.89	58.18 ± 20.66	< 0.001*

Data are expressed as mean ± standard deviation. *Statistically significant. TTE, transthoracic echocardiography; CCT, cardiac computed tomography; LVEF, left ventricular ejection fraction; LVEDVi, left ventricular end-diastolic volume index; LVESVi, left ventricular end-systolic volume index; LVSVi: left ventricular stroke volume index; LVCOi, left ventricular cardiac output index; LVMASS, left ventricular mass; LVMASSi, left ventricular mass index to body surface area.

### Left Atrial Strain and Volume Assessment

**FIGURE 3:**
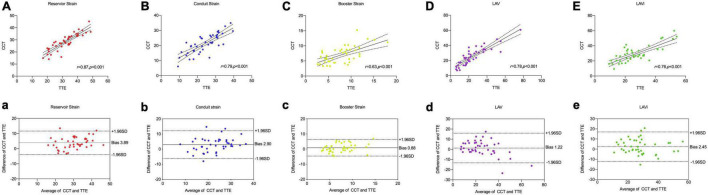
Comparison of LA strain and volume between CCT and TTE. Scatter plots and Bland–Altman analysis of LA reservoir strain **(A,a)** conduit strain **(B,b)**, booster strain **(C,c)**, LAV **(D,d)**, and LAVi **(E,e)**.

Table 4 and Figure 3 showed the correlation and Bland–Altman analysis for LA strain parameters and volume between CCT and TTE. LA strain and volume measurements showed good correlation and agreement between the two modalities (*r* = 0.63–0.87, *p* < 0.001), among them, the correlation of reservoir strain was the strongest (*r* = 0.87, *p* < 0.001), and the correlation of booster strain was relatively weak (*r* = 0.63, *p* < 0.001). All strain parameters of CCT were lower than these of TTE (reservoir strain: 28.37 ± 6.92 *vs.* 32.15 ± 8.15, respectively; conduit strain: 21.33 ± 6.46 *vs.* 24.23 ± 7.75, respectively; booster strain: 7.04 ± 2.74 *vs.* 7.92 ± 3.56). However, the volume parameters of CCT were higher than those of TTE (LAV: 29.60 ± 19.01 *vs.* 25.66 ± 17.60, respectively; LAVi: 30.36 ± 22.31 *vs.* 28.63 ± 19.25, respectively).

**TABLE 4:** Comparison of LA strain and volume parameters between CCT and TTE.

Measurement	CCT	TTE	Correlation		Bland–Altman	
	(Mean ± SD)	(Mean ± SD)	*r*-value	*p*-value	Bias	Limit of agreement
Reservoir strain (%)	28.37 ± 6.92	32.15 ± 8.15	0.87	<0.001*	3.89	−4.00 to 11.62
Conduit strain (%)	21.33 ± 6.46	24.23 ± 7.75	0.79	<0.001*	2.90	−6.32 to 12.12
Booster strain (%)	7.04 ± 2.74	7.92 ± 3.56	0.63	<0.001*	0.88	−4.64 to 6.41
LAV (ml)	29.60 ± 19.01	25.66 ± 17.60	0.78	<0.001*	1.22	−13.46 to 15.90
LAVi (ml/m2)	30.36 ± 22.31	28.63 ± 19.25	0.76	<0.001*	2.45	−12.13 to 17.02

Data are expressed as mean ± standard deviation. *Statistically significant. r, correlation coefficient; TTE, transthoracic echocardiography; CCT, cardiac computed tomography. LAV, left atrial volume; LAVi, left atrial volume indexed to body surface area.

### Univariate Associations of Left Atrial Strain With Left Atrial Volume and Left Ventricular Mass

**FIGURE 4:**
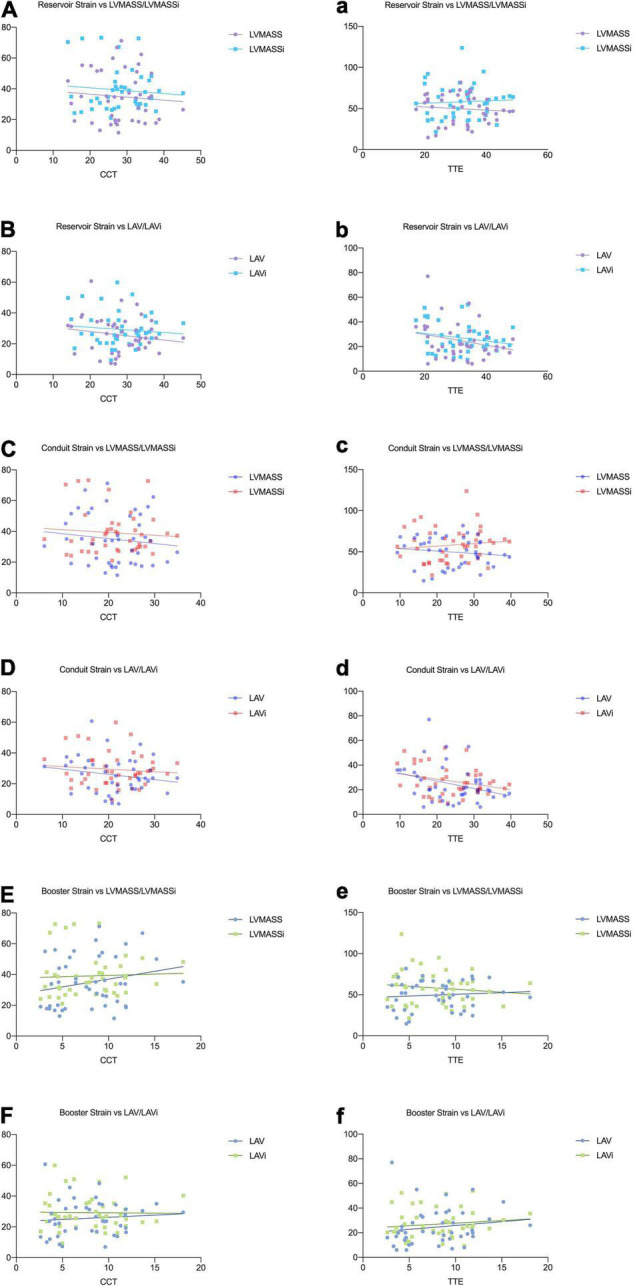
Relationships between the left atrial strain and the parameters of LA volume and LV mass in CCT **(A–F)** and TTE **(a–f)**.

Correlation analysis showed that reservoir and conduit strain were negatively correlated to LAV/LAVi and LVMASS/LVMASSi in both CCT and TTE. The booster strain was positively correlated to LAV/LAVi and LVMASS/LVMASSi (Table 5 and Figure 4).

**TABLE 5:** Correlations between left atrial strain and LA volume and LVMASS measurements of the CCT and TTE.

Variable of CCT	LVMASS		LVMASSi		LAV		LAVi	
	*R*	*p*	*r*	*p*	*r*	*p*	*r*	*p*
Reservoir strain	−0.08	0.603	−0.09	0.556	−0.32	0.038	−0.33	0.029
Conduit strain	−0.13	0.422	0.07	0.683	−0.33	0.033	−0.30	0.052
Booster strain	0.09	0.567	−0.03	0.826	−0.34	0.828	−0.14	0.372

**Variable of TTE**	**LVMASS**		**LVMASSi**		**LAV**		**LAVi**	
	***r***	***p***	***r***	***p***	***r***	***p***	***r***	***p***

Reservoir strain	−0.09	0.569	0.06	0.720	−0.30	0.055	−0.26	0.088
Conduit strain	−0.13	0.407	0.12	0.459	−0.35	0.021	−0.29	0.061
Booster strain	0.08	0.59	−0.12	0.431	−0.15	0.555	−0.03	0.874

*r, correlation coefficient; TTE, transthoracic echocardiography; CCT, cardiac computed tomography; LVMASS, left ventricular mass; LVMASSi, left ventricular mass index to body surface area; LAV, left atrial volume; LAVi, Left atrial volume indexed to body surface area.*

### Reproducibility Analysis

**FIGURE 5:**
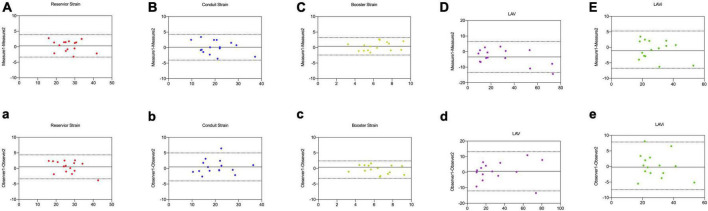
Intra-observer **(A–E)** and inter-observer agreement **(a–e)** for LA reservoir strain, conduit strain, booster strain, LAV, and LAVi measurements by CCT in 15 random studies.

Intra- and inter-observer reproducibility were presented in Table 6 and Figure 5. A major concern for using LA function as a reliable assessment was the inter- and intra-observer variability. In our study, CT-derived LA strain and volume measurements showed excellent intra- and inter-observer reproducibility using prototype software (*ICC* = 0.82–0.96), except the inter-observer reproducibility for booster strain (*ICC* = 0.78). Intra-observer reproducibility was slightly higher compared with inter-observer reproducibility.

**TABLE 6:** Intra-and inter-observer reproducibility of LA strain and volume parameters measured by CCT.

Parameters	Bias	Limit of agreement	*ICC*
**Intra-observer reproducibility (*n* = 15)**			
Reservoir strain (%)	0.28	−3.34 to 3.90	0.96
Conduit strain (%)	0.11	−4.04 to 4.25	0.95
Booster strain (%)	0.38	−2.43 to 3.21	0.82
LAV (ml)	−3.45	−13.45 to 6.53	0.96
LAVi (ml/m^2^)	−1.11	−6.78 to 5.25	0.93
**Inter-observer reproducibility (*n* = 15)**			
Reservoir strain (%)	0.48	−3.37 to 4.34	0.95
Conduit strain (%)	0.49	−4.02 to 4.99	0.94
Booster strain (%)	0.39	−3.21 to 2.43	0.78
LAV (ml)	0.52	−12.07 to 13.13	0.94
LAVi (ml/m^2^)	−0.21	−7.43 to 7.86	0.91

*LAV, left atrial volume; LAVi, left atrial volume indexed to body surface area.*

## Discussion

The present study showed that CCT was feasible for assessment of LA strain and volume in pediatric patients with CHD. According to our research, we found that LA strain and volume parameters measured by CCT had a good correlation and agreement with TTE; and CCT was a reliable method to assess LA strain and volume with good intra- and inter-observer reproducibility. These findings highlighted the importance of CCT in evaluating the LA strain and volume in some patients who had poor acoustic TTE windows, and was intolerant to or had contraindications to CMR.

It is worth noting that TTE is a reliable method to assess LA functional parameters, and several studies have demonstrated the utility of LA functional assessment using TTE (25, 26). However, the relative dependence on image quality and lower spatial resolution of TTE remains challenging in some pediatric patients who have poor acoustic windows, especially those with postoperative sternal deformity and thick bodies. What’s more, there are also important differences in individual examiners’ experiences (27). With the development of technology, CCT not only has great advantages in displaying anatomical structure as the result of excellent spatial resolution and imaging quality, but also can evaluate the cardiac function (28, 29). With the application of low-dose protocols and a short-scan time, the utility of CCT in pediatric patients has started to gain popularity (28). To our knowledge, there are few studies that have used CCT to evaluate LA function in pediatric patients. Thus, the present study was the first to compare LA strain and volume parameters between CCT and TTE in children with CHD.

Left atrial volume, in particular LAVi, has emerged as an important biomarker for adverse cardiac events in a variety of cardiovascular diseases and was the most sensitive parameter in predicting cardiovascular outcomes (30, 31). In our present study, TTE underestimated the LA volume compared with CCT, the measurements of LA volume by CCT showed moderate correlation and agreement with TTE. Similar results were also observed in some previous studies. Those findings regarded that due to geometric assumptions about LA shape and foreshortening of the LA cavity in the apical views, LA volume measured with TTE is often underestimated when compared with CCT (32, 33).

More recently, LA strain analysis has become one of the most interesting subjects in the assessment of LA function. The LA strain parameters have been shown to be helpful for the diagnosis of heart failure and be accurate predictors of clinical outcomes (8). One previous study considered that LA strain could be a more useful parameter to detect earlier LV diastolic alterations than LAVi in patients with preserved LVEF (12). Another study provided important insights regarding the potential usefulness and clinical relevance of adding LA strain to LAVi in the detection of LV diastolic dysfunction. They also found that the abnormal LA strain was significantly associated with worse NYHA functional class (14). Remarkably, Freed et al. found that the LA reservoir strain had better prognostic and discriminative utility when compared with the previously established LV longitudinal strain measurements (34).

As a newcomer technique, CCT is also increasingly applied to assess the LA stain. Hirasawa et al. (22) evaluated the agreement between STE and CCT for the measurement of LA longitudinal strain in patients prior to transcatheter aortic valve implantation, they found that CCT and STE had a good agreement and CCT may be an important adjuvant modality for assessing LA reservoir function in patients with severe AS. Similarly, Szilveszter et al. (21) also found a good correlation between CTA and echocardiography for the measurement of LA strain in patients following transcatheter aortic valve implantation (*n* = 28), and CTA provided accurate strain measurements with high reproducibility. Based on these aforementioned studies, our present study focused on the pediatric patients with CHD to explore more possibilities of CCT for the assessment of LA strain. The results of our study showed that CCT had a good correlation with TTE for the evaluation of LA strain (*r* = 0.63–0.87), and noted sufficient reproducibility. What’s more, our study found that the LA strain parameters of CCT were lower compared to TTE. This underestimation may be due to the software differences between the two modalities, TTE software includes the whole thickness of LA wall, and CCT represents the shortening of the endocardial boundary (22). On the other hand, this also may be explained by the lower temporal resolution of CCT. CCT had a temporal resolution of 17 frames/cardiac cycle in our study, while TTE had a temporal resolution of 24–30 frames/cardiac cycle.

As a complementary measurement tool, CCT is feasible for evaluating LA strain and volume in some pediatric patients who had a poor acoustic window or contradiction of CMR. Furthermore, the radiation risk of CT in children cannot be ignored. Although the real risks of CT remain unclear, the benefit of an appropriately indicated CT scan and improving the awareness of potentially harmful effects may far exceed the associated risks. In recent years, there are many studies on radiation dose reduction in pediatric patients, optimizing acquisition parameters is crucial to maintain and achieve acceptable image quality at the lowest possible radiation dose. At present, the dose reduction techniques included body-size-adapted protocol, low tube voltage, tube current modulation, and iterative reconstruction algorithm in daily clinical practice for pediatric CT (35, 36). In our group, we also used the low-dose scanning techniques to minimize the radiation dose. The effective dose (2.23 mSv) was lower than previous studies about CCT in pediatric patients with CHD (37, 38).

There were several limitations in the present study that should be acknowledged. Firstly, this was a single center with relatively modest sample size and the data should be interpreted with caution. Secondly, the different dedicated software for LA strain analysis in our study may generate biases in comparison of CCT and TTE. Finally, we only evaluated the feasibility and reproducibility of CCT-derived LA strain measurements in the present study. The potential importance and clinical utility of LA function in CHD are required for further investigations in the future.

## Conclusion

In conclusion, CCT can regard as an accepted method for measuring LA strain and volume with good correlation and high reproducibility. As a complementary modality, CCT plays an important role in the evaluation of LA function in some pediatric patients who have limitations with TTE or CMR.

## Data Availability Statement

The original contributions presented in this study are included in the article/supplementary material, further inquiries can be directed to the corresponding authors.

## Ethics Statement

The studies involving human participants were reviewed and approved by the Shanghai Children’s Medical Center Ethics Committee. Written informed consent to participate in this study was provided by the participants or their legal guardian/next of kin.

## Author Contributions

W-HX, L-JC, L-WH, Q-HF, and Y-MZ were involved in the study design. W-HX, L-JC, and Y-MZ drafted the manuscript and were involved in data analysis. L-WH, R-ZO, CG, A-MS, QW, H-SQ, and Y-QZ participated in data acquisition. W-HX and L-JC performed the statistical analysis. All authors made appropriate contributions to the manuscript, critically reviewed, and approved the final manuscript.

## Conflict of Interest

The authors declare that the research was conducted in the absence of any commercial or financial relationships that could be construed as a potential conflict of interest.

## Publisher’s Note

All claims expressed in this article are solely those of the authors and do not necessarily represent those of their affiliated organizations, or those of the publisher, the editors and the reviewers. Any product that may be evaluated in this article, or claim that may be made by its manufacturer, is not guaranteed or endorsed by the publisher.
